# Interaction of ALK Inhibitors with Polyspecific Organic Cation Transporters and the Impact of Substrate-Dependent Inhibition on the Prediction of Drug–Drug Interactions

**DOI:** 10.3390/pharmaceutics15092312

**Published:** 2023-09-13

**Authors:** Yik Pui Tsang, Antonio Jesús López Quiñones, Letícia Salvador Vieira, Joanne Wang

**Affiliations:** Department of Pharmaceutics, University of Washington, Seattle, WA 98195, USA; a6245912@uw.edu (Y.P.T.); alopezq@uw.edu (A.J.L.Q.); lsvieira@uw.edu (L.S.V.)

**Keywords:** drug transport, drug–drug interactions, organic cation transporters, tyrosine kinase inhibitors, anaplastic lymphoma kinase, transporter-mediated drug disposition

## Abstract

Small molecules targeting aberrant anaplastic lymphoma kinase (ALK) are active against ALK-positive non-small-cell lung cancers and neuroblastoma. Several targeted tyrosine kinase inhibitors (TKIs) have been shown to interact with polyspecific organic cation transporters (pOCTs), raising concerns about potential drug–drug interactions (DDIs). The purpose of this study was to assess the interaction of ALK inhibitors with pOCTs and the impact of substrate-dependent inhibition on the prediction of DDIs. Inhibition assays were conducted in transporter-overexpressing cells using meta-iodobenzylguanidine (mIBG), metformin, or 1-methyl-4-phenylpyridinium (MPP+) as the substrate. The half-maximal inhibitory concentrations (IC_50_) of brigatinib and crizotinib for the substrates tested were used to predict their potential for in vivo transporter mediated DDIs. Here, we show that the inhibition potencies of brigatinib and crizotinib on pOCTs are isoform- and substrate-dependent. Human OCT3 (hOCT3) and multidrug and toxin extrusion protein 1 (hMATE1) were highly sensitive to inhibition by brigatinib and crizotinib for all three tested substrates. Apart from hMATE1, substrate-dependent inhibition was observed for all other transporters with varying degrees of dependency; hOCT1 inhibition showed the greatest substrate dependency, with differences in IC_50_ values of up to 22-fold across the tested substrates, followed by hOCT2 and hMATE2-K, with differences in IC_50_ values of up to 16- and 12-fold, respectively. Conversely, hOCT3 inhibition only showed a moderate substrate dependency (IC_50_ variance < 4.8). Among the substrates used, metformin was consistently shown to be the most sensitive substrate, followed by mIBG and MPP+. Pre-incubation of ALK inhibitors had little impact on their potencies toward hOCT2 and hMATE1. Our results underscore the complexity of the interactions between substrates and the inhibitors of pOCTs and have important implications for the clinical use of ALK inhibitors and their DDI predictions.

## 1. Introduction

Anaplastic lymphoma kinase (ALK) is a neural receptor tyrosine kinase (RTK) in the insulin receptor superfamily that is predominantly expressed in the central and peripheral nervous system [1]. Mutations in the gene encoding ALK have been linked to multiple cancers, including the nucleophosmin (NPM1)-ALK fusion oncoprotein observed in anaplastic large-cell lymphoma (ALCL) and the echinoderm microtubule-associated protein-like 4 (EML4)-ALK in non-small-cell lung cancer (NSCLC), which significantly contribute to tumor cell cycle progression, migration, and evasion of apoptosis [2,3,4,5,6,7]. Furthermore, mutations in the intracellular kinase domain and gene copy amplifications resulting in aberrant ALK activity have also been reported, specifically in neuroblastoma (NBL) and NSCLC, respectively [1,4,8]. Given its pivotal role in these cancers, ALK was identified as an important therapeutic target. Multiple ALK inhibitors have since then been developed, including crizotinib, a tyrosine kinase inhibitor (TKI) approved by the US Food and Drug Administration (FDA) in 2011 and indicated for ALK-positive NSCLC [9]. Crizotinib is currently undergoing phase-3 clinical trials for NBL as an addition to standard chemotherapy [10]. Several other next-generation ALK inhibitors, such as brigatinib and lorlatinib, have been approved by the FDA for NSCLC [11]. Although none has been approved by the FDA for NBL, a recent phase-1 trial showed promising efficacy of lorlatinib against high-risk ALK-driven NBL [12].

Polyspecific organic cation transporters (pOCTs) belong to the family of human solute carrier (SLC) transporters, which facilitate the cellular influx and efflux of different drug molecules and toxins, as well as many endogenous organic cations [13,14]. This group contains the electrogenic-membrane-potential-driven organic cation transporters 1 to 3 (hOCT1, 2, 3) and the transmembrane-proton-gradient-driven multidrug and toxin extrusion proteins 1 and 2-K (hMATE1, 2-K). In humans, OCT1 is mostly expressed in the liver and localized basolaterally in hepatocytes, which facilitates cellular organic cation uptake [13]. OCT2 and MATE1/2-K are predominantly and respectively expressed on the basolateral and apical membrane of the renal proximal tubular epithelial cells (PTECs) in the kidneys, facilitating renal tubular secretion of organic cations [13,14,15]. OCT3 is broadly distributed throughout the body with relatively greater expression in the salivary glands, placenta, adrenal glands, skeletal muscle, and heart [16,17,18,19,20], and our group has previously demonstrated its role in drug uptake into some of these tissues [17,21]. Several commonly used cancer chemotherapeutic agents, including certain platinum compounds, such as cisplatin and oxaliplatin, as well as some anthracyclines, such as doxorubicin, are substrates for OCTs and/or MATEs [22,23,24]. Thus, these transporters can potentially participate in not only the tumor uptake of anticancer agents, but also in their disposition in normal tissues. For instance, our recent study suggested that the distribution of meta-iodobenzylguanidine (mIBG), a radiopharmaceutical used for targeted imaging and treatment of neuroendocrine tumors, is mediated by hOCT1 and 3 in normal tissues, while hOCT2 and hMATE1/2-K play a role in its renal elimination [25].

Drug–drug interactions (DDIs) involving transporters are of considerable clinical concern, as alterations in transporter function can influence the disposition, efficacy, and safety of the impacted drugs, often referred to as ‘victim drugs’. Numerous reported DDIs have been ascribed to the inhibition of hOCT2 and hMATEs in the kidneys [15,26,27]. Furthermore, OCT1 and 3 are involved in the hepatic clearance of cationic xenobiotics and endogenous substances, as well as in the intestinal absorption of many orally administered cationic drugs [17,28]. These findings highlight the importance of assessing clinically relevant DDIs mediated by transporters during drug development [13]. According to the guidelines published by the FDA and the International Transporter Consortium (ITC), if a new molecular entity (NME) is an in vitro inhibitor of certain relevant transporters and the ratio of its unbound maximal plasma concentration (C_max_) to its half-maximal inhibitory concentration (IC_50_) is greater than 0.1, further in vivo DDI assessment is recommended [13,29]. For pOCTs, most in vitro screens are conducted with well-established probe substrates such as metformin or 1-methyl-4-phenylpyridinium (MPP+). However, emerging evidence suggests that substrate-dependent inhibition can occur with pOCTs, possibly due to the interactions of inhibitors and substrates with multiple binding sites on the transporter protein [30]. For instance, our group previously observed that cimetidine, an OCT2 inhibitor, showed a 10-fold higher potency with atenolol as a substrate compared with metformin [31]. Another group also showed that inhibition of hOCT2 is highly substrate-dependent using cationic ionic liquids [32]. Evidently, hMATE1 is less impacted by substrate choice compared with hOCT2 [31,33], although conflicting results have been reported [32]. Currently, little information is available on substrate-dependent inhibition for hOCT1, hOCT3, and hMATE2-K.

Previous studies have shown that TKIs, including some ALK inhibitors, interact with pOCTs [34,35,36]. However, their reported inhibitory profiles and selected substrates vary among studies. Furthermore, these studies used either experimental compounds (e.g., 4–4-dimethylaminostyryl-*N*-methylpyridinium (ASP+) and MPP+) or non-cancer drugs (e.g., metformin) as substrates, which may not be suitable to predict pOCT-mediated ALK drug interactions due to substrate-dependent inhibition. Here, we systematically assessed the in vitro inhibitory effects of three selected ALK inhibitors, namely lorlatinib, crizotinib, and brigatinib, on hOCT1 to 3- and hMATE1/2-K-mediated transport of mIBG, which has a high chance of being used in combination with ALK inhibitors to diagnose and treat NBL and other neuroendocrine cancers [12]. The three ALK inhibitors were selected due to their significant potential in clinical translation to treat ALK-driven NBL, as (1) all three ALK inhibitors have been approved by the FDA for treating NSCLC and (2) all have demonstrated in vitro and in vivo activity against ALK-positive NBL cells [12,37,38]. Erlotinib, despite being an epidermal growth factor receptor (EGFR)-targeting TKI, was also included in the initial screening process due to its potential to treat NBL when combined with a B-cell lymphoma 2 antagonist [39]. Potent transporter inhibitors were further investigated by performing dose-dependent inhibition studies and determining their IC_50_ values. These studies were then performed using metformin or MPP+ as substrates to investigate the influence of probe substrate selection on inhibitor interactions with the transporters. Prediction of the in vivo inhibition of OCTs and MATEs for potent TKIs was also performed using the criteria defined and recommended by the FDA. Finally, while time-dependent inhibition has previously been observed in some transporters, such as the organic anion-transporting polypeptides (OATP) 1B1 and 3 [40,41], it remains unclear whether this holds true for pOCTs. Therefore, the effects of preincubation on the inhibition potencies of TKIs were also investigated with the major renal transporters OCT2 and MATE1.

## 2. Materials and Methods

### 2.1. Chemicals and Reagents

MIBG, crizotinib, lorlatinib, and glyburide were purchased from Sigma-Aldrich (St. Louis, MO, USA). Erlotinib was kindly provided by Dr. Rheem Totah. Brigatinib was purchased from MedChemExpress (Monmouth Junction, NJ, USA). [^14^C]metformin (112 mCi/mmol) and [^3^H]1-methyl-4-phenylpyridinium (MPP+, 80 Ci/mmol) were purchased from American Radiolabeled Chemicals, Inc. (St. Louis, MO, USA). Acetonitrile (ACN) and formic acid (liquid chromatography with tandem mass spectrometry (LC-MS/MS)-grade) were obtained from Thermo Fisher Scientific (Rockford, IL, USA). Reagents for cell culture were purchased from Invitrogen (Carlsbad, CA, USA).

### 2.2. Cell Culture

The cell culture protocol was as previously described [20,31]. In brief, Flp-In human embryonic kidney (HEK) 293 cells stably transfected with hOCT1 to 3, hMATE1/2-K, and empty pcDNA5/FRT vector (mock cells), previously established in our laboratory [20,31], were maintained and cultured in Dulbecco’s modified Eagle’s medium enriched with 10% fetal bovine serum (FBS), 2 mM L-glutamine, 100 U/mL penicillin/100 μg/mL streptomycin (1X P/S), and 150 μg/mL hygromycin B. Cells were cultured in a 37 °C incubator with 5% CO_2._ Culture flasks were coated with 0.01% poly-D-lysine (PDL) to enhance the attachment of cells.

### 2.3. Inhibition Screening and Uptake Assays in HEK293 Cells

Uptake assays were performed as previously described [25]. In brief, cells were seeded on PDL-coated 96-well plates and grown to at least 90% confluency. On the day of the experiment, cells were first washed with 200 μL of prewarmed Hank’s balanced salt solution (HBSS) buffer (Thermo Fisher Scientific, Rockford, IL, USA) 3 times. For hMATEs-transfected cells, the pH of the HBSS buffer was adjusted from 7.4 to 8.0 to facilitate inward substrate uptake by establishing an outward-facing proton gradient. Measurement was carried out after an incubation period of 2 min at 37 °C at an mIBG concentration of 1 µM. Experiments began with the addition of 100 μL of HBSS buffer in the presence or absence of an inhibitor. For the initial screening, 20 μM of the inhibitor was used. For the determination of IC_50_ values, a range of 12 concentrations, generated through 1:1 serial dilution from 100 μM (100, 50, 25, 12.5, 6.25, 3.12, 1.56, 0.78, 0.39, 0.20, and 0 μM), was used. The percentages of organic solvent in the incubation buffer with substrates and inhibitors were normalized accordingly and kept below 0.5%. Uptake was stopped by aspirating the incubation buffer and washing the cells with 200 μL of ice-cold HBSS at least 3 times. The cells were then lysed using 200 μL of 10% ACN containing 50 nM glyburide (internal standard) per well, and 20 μL of the lysates were diluted 1:10 in 10% ACN for mIBG quantification. For [^14^C]metformin and [^3^H]MPP+, assays were performed in the same manner as for mIBG, but the cells were instead lysed with 100 μL of 1 M NaOH for 1 h at 37 °C and subsequently neutralized with 100 μL of 1 M HCl, of which 150 μL of the total cell lysate was used for total radioactivity analysis using a Tri-Carb Liquid Scintillation Counter (Perkin Elmer, Waltham, MA, USA). For preincubation assays, cells were preincubated with the TKIs in HBSS for 30 min before the initiation of substrate uptake.

### 2.4. Quantification of mIBG by LC-MS/MS

Due to concerns about rapid iodine isotope decay (t_1/2_ ~ 13 h for ^123^I and 8 days for ^131^I), nonradioactive mIBG was used and quantified by LC-MS/MS as previously described [25]. Briefly, an Agilent 6410 Triple Quad mass spectrometer coupled with an Agilent 1290 series HPLC system (Agilent Technologies, Santa Clara, CA, USA), operated in positive electrospray ionization mode (ESI+), was used for the mIBG quantification. Ten microliters of the diluted cell lysate samples at 4 °C with internal standards were injected through a Zorbax Eclipse Plus C18 column (2.1 mm × 50 mm, 1.8 µm) (Agilent Technologies, Santa Clara, CA, USA) maintained at room temperature. For the mobile phase, 0.1% formic acid in water (A) and 0.1% formic acid in acetonitrile (B) was used. A 5 min ballistic gradient detailed as follows was used for the compound elution: 3% B from 0 to 0.1 min, 3% B to 90% B from 0.1 to 2 min, 90% B from 2 to 3 min, 90% B to 3% B from 3 to 3.1 min, and 3% B from 3.1 to 5 min. The mass-to-charge (*m*/*z*) transitions used were 276.1 → 217.0 for mIBG and 494.1 → 369.0 for glyburide.

### 2.5. Data Analysis

The uptake experiments were conducted in technical triplicate and repeated independently 3 times with cells from different passages. For LC-MS/MS, instrument navigation and raw data processing and analysis were performed with Analyst software 1.6 (AB Sciex, Framingham, MA, USA). The lower limit of quantification (LLOQ) of mIBG was 0.1 nM, and the calibration curve was linear up to 8 μM. Non-linear regression was used to fit the data on GraphPad Prism 7.0 (GraphPad Software Inc., La Jolla, CA, USA) to generate graphs and calculate parameters. For the dose-dependent inhibition assay, the IC_50_ values of inhibitors were calculated with the equation below:

(1) $$v=\mathrm{Bottom}+\frac{\mathrm{Top}-\mathrm{Bottom}}{1+{\left(\frac{\left[I\right]}{{\mathrm{IC}}_{50}}\right)}^{h}}$$

where v is the rate of substrate uptake with inhibitor, Bottom is the baseline value at 100% inhibition, Top is the rate of substrate uptake without inhibitor, [I] is the inhibitor concentration, IC_50_ is the half-maximal inhibitory concentration, and h is the Hill coefficient.

## 3. Results

### 3.1. Inhibitory Screening of Selected TKIs on the Uptake of mIBG by hOCT1-3 and hMATE1/2-K

**Figure 1:**
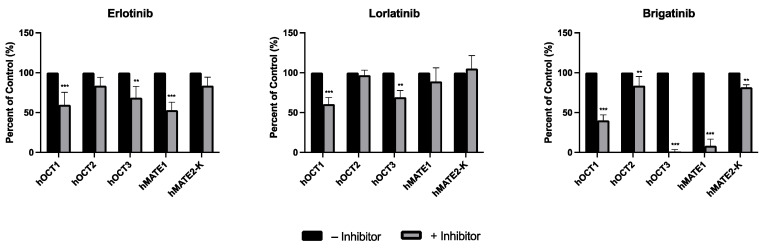
Inhibitory effects of erlotinib, lorlatinib, and brigatinib on meta-iodobenzylguanidine (mIBG) uptake mediated by human organic cation transporter 1 to 3 (hOCT1-3) and human multidrug and toxin extrusion protein (hMATE1/2-K). Intracellular accumulation of mIBG in human embryonic kidney 293 (HEK293) cells transfected with an empty vector (mock cells), hOCT1-3, and hMATE1/2-K, with and without 20 µM of the selected inhibitors, was quantified. Measurement was carried out after an incubation period of 2 min at 37 °C, at an mIBG concentration of 1 µM. The uptake of mIBG mediated specifically by transporters was calculated by subtracting the amount of mIBG (measured in pmol per mg of total protein) in mock cells from the amount of mIBG measured in transporter-transfected cells. Data are expressed as means ± SD from three independent experiments as a percentage of mIBG uptake without an inhibitor (control), and the uptake of mIBG with an inhibitor is compared with the control. Statistical significance was determined by performing an unpaired Student’s *t*-test with a Bonferroni correction for multiple comparisons (** *p* < 0.01; *** *p* < 0.001).

Erlotinib, lorlatinib, and brigatinib were used at a relatively low concentration of 20 μM for the initial screening to determine their inhibitory effects on the transport of mIBG by hOCT1-3 and hMATE1/2-K. Data for crizotinib are not shown here, as it was screened previously [25]. At the tested concentration, erlotinib and lorlatinib did not inhibit mIBG uptake by any of the transporters by more than 50% (Figure 1). In contrast, brigatinib potently inhibited mIBG uptake by hOCT3 and hMATE1 by more than 80% and by hOCT1 by more than 50%. 

### 3.2. Dose-Dependent Inhibition Profile and IC_50_ Determinations of Selected TKIs on the Uptake of mIBG Mediated by hOCT1-3 and hMATE1/2-K

**Figure 2:**
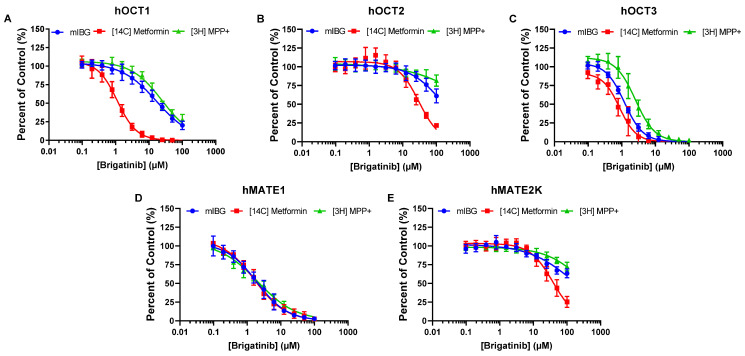
Dose-dependent inhibition of hOCT1-3 and hMATE1/2-K by brigatinib using mIBG, metformin, and 1-methyl-4-phenylpyridinium (MPP+) as substrates. Uptake of 1 µM mIBG, 8.9 µM [^14^C]metformin, and 1 µM [^3^H] MPP+ in the absence and presence of brigatinib was measured in vector, hOCT1 (**A**), hOCT2 (**B**), hOCT3 (**C**), hMATE1 (**D**), and hMATE2-K (**E**) expressing HEK293 cells after 2 min of incubation at 37 °C. The uptake of substrate mediated specifically by transporters was calculated by subtracting the amount of substrate (measured in pmol per mg of total protein) in mock cells from the amount of substrate measured in transporter-transfected cells. Data are expressed as means ± SD from three independent experiments as a percentage of substrate uptake without brigatinib (control).

**Figure 3:**
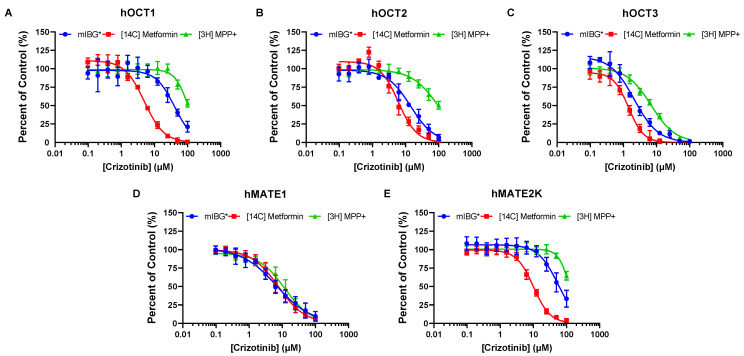
Dose-dependent inhibition of hOCT1-3 and hMATE1/2-K by crizotinib using mIBG, metformin, and MPP+ as substrates. Data were measured and presented in the same manner as described in Figure 2. Crizotinib inhibition of hOCT1-3-mediated mIBG uptake was previously assessed in our lab and is referenced here for comparison (*) [25].

Subsequent dose-dependent inhibition studies were conducted, and the IC_50_ values for brigatinib and crizotinib were determined, with substrates being used at concentrations much lower than their corresponding K_m_ values. Brigatinib inhibition of hOCT3 was 14-fold and at least 100-fold more potent than that of hOCT1 and hOCT2, respectively, and its inhibition of hMATE1 was at least 50-fold more potent than of hMATE2-K (Figure 2 and Table 1). The IC_50_ values determined for hMATE1 were eight-fold lower than those for hMATE2-K, indicating that crizotinib is more selective towards hMATE1 (Figure 3D,E and Table 1).

### 3.3. Brigatinib and Crizotinib Inhibition of hOCTs and hMATE2-K Is Highly Dependent on the Substrates Used

Dose-dependent inhibition studies of hOCTs and hMATEs were performed with crizotinib and brigatinib as inhibitors and metformin and MPP+ as substrates. At the tested concentrations, significant substrate-dependent inhibition was observed for both brigatinib and crizotinib (Figure 2 and Table 1). Metformin was shown to be the most sensitive substrate to inhibition among the three substrates, followed by mIBG and MPP+. The dependency was most pronounced in hOCT1, where brigatinib was approximately 15-fold and 22-fold more potent and crizotinib was approximately 8-fold and 21-fold more potent when metformin was used as the substrate rather than mIBG or MPP+, respectively (Table 1). Brigatinib was not a strong inhibitor of hOCT2. The IC_50_ curves of hOCT2 for brigatinib were not completely captured, and the IC_50_ values cannot be accurately determined for MPP+. Nevertheless, substrate-dependent inhibition was apparent (Figure 2B). Crizotinib was approximately 2-fold and 16-fold more potent towards hOCT2 when metformin was used as the substrate rather than mIBG or MPP+, respectively (Figure 3 and Table 1). Substrate-dependent inhibition for hOCT3 was the least pronounced among the three hOCTs, with differences in IC_50_ values varying up to 3-fold for brigatinib and 5-fold for crizotinib. For hMATE2-K, IC_50_ values for mIBG and MPP+ could not be accurately determined due to weak inhibition. Regardless, substrate-dependent inhibition was observed. Brigatinib was approximately 4-fold and 10-fold more potent and crizotinib was approximately 5-fold and 12-fold more potent when metformin was used as the substrate rather than mIBG or MPP+, respectively (Table 1). Interestingly, the influence of substrate was minimal for hMATE1. The differences in IC_50_ values were below two-fold for both brigatinib and crizotinib across all three tested substrates (Figure 2 and Figure 3 and Table 1).

### 3.4. Impact of Substrate-Dependent Inhibition on the Prediction of DDI Potential for Brigatinib and Crizotinib

To assess whether the observed substrate-dependent inhibition by brigatinib and crizotinib could lead to different outcomes for the FDA-recommended in vivo DDI assessment, the unbound C_max_/IC_50_ values were calculated for each substrate. For brigatinib, varying in vivo study recommendations were noted: for hOCT1, it was a ‘Yes’ for metformin, but a ‘No’ for mIBG and MPP+ (Table 2). While the inhibition of hOCT3 and hMATE1 by brigatinib exhibited less pronounced substrate-dependent inhibition, all unbound C_max_/IC_50_ values exceeded 0.1, resulting in a consensus in recommendations across all substrates for in vivo DDI studies. For hOCT2 and hMATE2-K, the unbound plasma concentrations of brigatinib might be insufficient to elicit an in vivo inhibitory effect, but even though the recommendations obtained are the same for both transporters across the three substrates, the unbound C_max_/IC_50_ values are quite different. Similarly, the unbound plasma concentrations of crizotinib might not be sufficient to inhibit hOCT2 or hMATE2-K in vivo, but the unbound C_max_/IC_50_ values for hOCT2 and hMATE2-K are significantly different among the three substrates (Table 3). These findings suggest that substrate-dependent inhibition can potentially influence the DDI prediction outcomes for hOCT1, hOCT2, and hMATE2-K.

### 3.5. Effect of Preincubation on the Inhibitory Effects of Brigatinib and Crizotinib on hOCT2 and hMATE1

**Figure 4:**
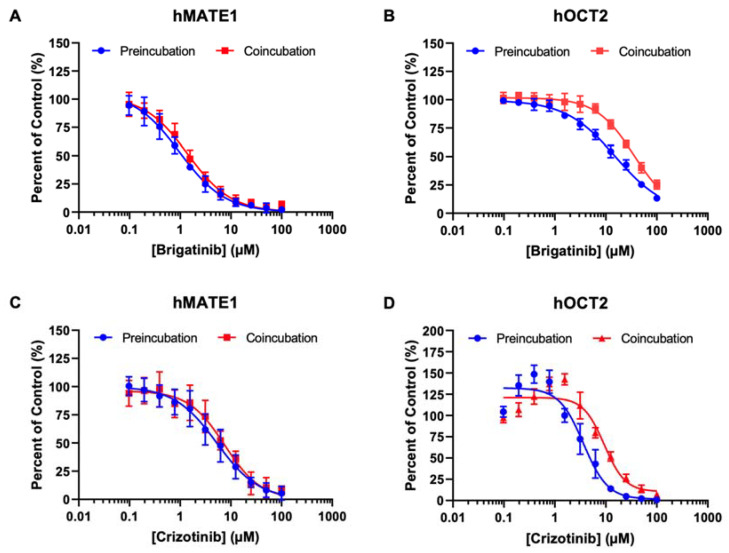
The impact of preincubation on the inhibitory potencies of brigatinib and crizotinib on the uptake of [^14^C] metformin by hMATE1 (**A**,**C**) and hOCT2 (**B**,**D**). Uptake of 8.9 µM [^14^C] metformin in the absence and presence of brigatinib and crizotinib was measured in vector, hMATE1 (**A**,**C**) and hOCT2 (**B**,**D**) expressing HEK293 cells after 2 min of incubation at 37 °C following a preincubation period with the inhibitor for 30 min at 37 °C. The uptake of substrate mediated specifically by transporters was calculated by subtracting the amount of substrate (measured in pmol per mg of total protein) in mock cells from the amount of substrate measured in transporter-transfected cells. Data are expressed as means ± SD from three independent experiments as a percentage of substrate uptake without brigatinib or crizotinib (control).

In clinical settings, an inhibitor drug might be administered to patients for a period before the victim drug (i.e., substrate) is given. While time-dependent inhibition has been previously observed in some transporters, such as the organic anion-transporting polypeptides (OATP) 1B1 and 3 [40,41], it remains unclear whether this also applies to pOCTs. Since hOCT2 and hMATE1 are the two major cation transporters that facilitate cationic drug elimination in the kidneys, we investigated how preincubation influenced the inhibition potencies of brigatinib and crizotinib on metformin uptake mediated by these transporters. Although a 30 min preincubation slightly increased the inhibitory potency of brigatinib and crizotinib for hOCT2, the increase was only around two-fold (Figure 4 and Table 4). The impact of preincubation on hMATE1 was minimal. These data suggest that pre-incubation has little impact on the inhibition potencies of ALK inhibitors for hOCT2 and hMATE1.

## 4. Discussion

Small molecules targeting aberrant ALK have been proven effective against ALK-positive NSCLC and NBL. These ALK inhibitors are often used alongside standard chemotherapy or other drugs to combat resistance, which subsequently raises concerns about potential DDIs. Here, we investigated the interactions between selected ALK inhibitors and pOCTs and determined whether their inhibition of hOCT1-3 and hMATE1/2-K was substrate- and isoform-dependent. Our results reveal that brigatinib and crizotinib potently and selectively inhibit hOCTs and hMATEs in an isoform- and substrate-dependent manner. Both hOCT3 and hMATE1 exhibit high sensitivity towards inhibition by brigatinib and crizotinib, regardless of the substrate used. Aside from hMATE1, substrate-dependent inhibition was observed for all other transporters, with varying degrees of dependency. These findings have multiple implications for the clinical use of ALK inhibitors in cancer patients and for in vitro to in vivo DDI predictions for pOCTs.

Radiolabeled mIBG serves as a therapeutic and diagnostic agent for neuroendocrine tumors, including NBL. Beyond tumor cells, mIBG is strongly accumulated in the heart, liver, and salivary glands, which can lead to unwanted toxicity in these normal tissues [42,43]. Our group previously demonstrated that mIBG uptake is facilitated by pOCTs, and these transporters play a crucial role in mIBG distribution into normal tissues [25]. Notably, OCT3 has been identified as the primary transporter facilitating mIBG into the heart and salivary glands [44]. We and others have thus proposed that inhibition of OCT3 could be a viable clinical strategy to improve the safety and tumor selectivity of mIBG in cancer patients [25,45,46]. Here, we showed that brigatinib exhibits potent and preferential in vitro inhibition of hOCT3 (Figure 2 and Table 1). While crizotinib has also been identified as a selective hOCT3 inhibitor [25], its unbound plasma concentrations might not be enough to inhibit hOCT3 in vivo, as predicted by its unbound C_max_/IC_50_ ratio (Table 3). Nonetheless, considering that ALK inhibitors have proven effective against high-risk ALK-positive NBL, and given that mIBG is taken up by NBL tumor cells via the norepinephrine transporter (NET) [47], combination therapy with mIBG and brigatinib could potentially lead to improved therapeutic outcomes.

Metformin, an oral hypoglycemic drug, is a primary treatment for type 2 diabetes, a condition that might share certain risk factors with cancer [48]. It has been shown that metformin, in combination with TKIs, yielded synergistic results in treating patients with advanced NSCLC who also have type 2 diabetes [49]. Ongoing clinical trials are investigating this combination (NCT03071705), suggesting that patients might potentially undergo combination therapy with TKIs and metformin. In the context of DDIs, this could have significant pharmacokinetic (PK) and pharmacodynamic (PD) implications. Metformin is not metabolized in the liver and is mostly eliminated unchanged in the urine through active tubular secretion facilitated by OCT2 and MATE1/2-K [50]. Therefore, concomitant administration of TKIs that potently inhibit OCT2 and/or MATE1/2-K with metformin could potentially reduce its renal clearance and change its PK profile. Additionally, metformin exerts its PD effects in the liver by lowering excessive glucose production via a reduction in gluconeogenesis [51], a process that is primarily rate-determined by the hepatic OCT1-mediated uptake of metformin. Thus, co-administration of metformin with TKIs that are also potent OCT1 inhibitors, such as brigatinib (Figure 2), might result in reduced efficacy of metformin and further complicate existing medical conditions.

Although both metformin and MPP+ are recommended by the ITC and the FDA as probe substrates for evaluating transporter-mediated DDIs toward OCTs and MATEs, substrate-dependent inhibition has been reported in the literature [30]. A previous study from our group indicated that with atenolol as a substrate instead of metformin, cimetidine acts as a more potent inhibitor towards hOCT2 [31]. To further investigate this phenomenon, we compared the inhibition profiles of crizotinib and brigatinib towards OCTs and MATEs with the three different substrates. Our data showed that the inhibition of hOCT1-3 and hMATE2-K by both crizotinib and brigatinib is substrate-dependent. Interestingly, hOCT1 showed the greatest substrate dependency, followed by hOCT2 and hMATE2-K. Conversely, substrate-dependent inhibition was relatively moderate for hOCT3. Among the substrates used, metformin was consistently shown to be the most sensitive to inhibition, followed by mIBG and MPP+ (Figure 2 and Figure 3). This is consistent with previously reported data from the literature and further supports the conclusion that substrate and inhibitor interactions are not restricted to a single binding site for pOCTs [30]. Instead, there are unique ligand interactions occurring at multiple high- or low-affinity cation binding sites in addition to allosteric interactions [52,53]. Consequently, the in vitro inhibition potency of a potential new inhibitor under investigation might be considerably influenced by the selected probe substrate and could lead to inconsistent recommendations for conducting in vivo DDI studies, as shown in Table 4. Therefore, it is important to consider and utilize not only one probe substrate, but two or more clinically relevant substrates for transporters that show significant substrate-dependent inhibition, as recommended by the FDA [29].

Unlike other pOCTs, the inhibition of hMATE1 by crizotinib and brigatinib is not influenced by the substrate (Figure 2 and Figure 3 and Table 1). This is consistent with previous findings from our group showing that cimetidine, pyrimethamine, and carvedilol all inhibited hMATE1-mediated uptake of atenolol or metformin with similar potencies [31]. Additionally, these results also agree with a study by Martínez-Guerrero et al. [33], where they utilized four structurally distinct organic cation substrates and showed that the identity of the substrate does not exert a significant influence on inhibitor interactions with hMATE1. Interestingly, a study conducted previously by the same group showed contradictory results suggesting that MATE1 inhibition is substrate-dependent [32]. Nonetheless, due to the structural diversity of TKIs and the complexity of ligand interactions with OCTs and MATEs, extending this result and conclusion from crizotinib and brigatinib to other TKIs for hMATE1 inhibition may not be appropriate. This stands as one major limitation of our study and further investigations are required with additional TKIs and structurally diverse substrates to evaluate whether inhibition of hMATE1 is independent of the substrate in use. 

Time-dependent inhibition has been observed previously with OATP1B1 and 3 [40,41]. Clinically, an inhibitor or perpetrator may be administered for an extended period to attain steady-state concentrations in the system before a substrate (i.e., the victim drug) is given [54]. Therefore, there may be an underestimation of the inhibitory potential of test compounds in in vitro transporter assays if the substrate and the inhibitor are coincubated, which only allows simultaneous and direct interaction at the substrate binding site. For renal transporters, this is especially important, since they represent a major pathway for systemic drug elimination [27]. Our data show that preincubation of brigatinib or crizotinib had a minimal effect on their inhibition potencies for hMATE1 (Figure 4 and Table 4). Nonetheless, preincubation appears to trend towards a more potent effect for hOCT2. Further in-depth studies are needed to elucidate the impact of preincubation on inhibition potencies for pOCTs.

In summary, our results show significant substrate-dependent inhibition of hOCT1-3 and hMATE2-K, but not of hMATE1, and demonstrate the importance of probe substrate choice in the prediction of in vivo DDIs with ALK inhibitors. These results underscore the complex mechanisms of the interactions between substrates and inhibitors of pOCTs and provide evidence that warrants further investigation of potential transporter-mediated DDIs between TKIs and clinically relevant victim drugs. 

## Figures and Tables

**Table 1:** Half-maximal inhibitory concentrations (IC_50_), shown as means ± SD from three independent experiments, of brigatinib and crizotinib for hOCT1-3- and hMATE1/2-K-mediated metformin, mIBG, and MPP+ uptake.

	IC_50_ (µM)					
	Brigatinib			Crizotinib		
	Metformin	mIBG	MPP+	Metformin	mIBG	MPP+
hOCT1	1.12 ± 0.04	17.1 ± 0.6	25.1 ± 1.2	4.98 ± 0.15	40.3 ± 5.5 ^1^	105 ± 3
hOCT2	30.2 ± 1.5	157 ± 11	>300	6.07 ± 0.34	14.3 ± 1.7 ^1^	98.1 ± 4.8
hOCT3	0.85 ± 0.07	1.20 ± 0.03	2.54 ± 0.14	1.43 ± 0.11	2.14 ± 0.25 ^1^	6.86 ± 0.20
hMATE1	1.67 ± 0.14	1.93 ± 0.15	2.03 ± 0.24	7.60 ± 0.26	6.73 ± 0.39	11.8 ± 0.4
hMATE2-K	38.3 ± 1.7	172 ± 17	>300	10.1 ± 0.3	55.4 ± 2.3	125 ± 4

^1^ IC_50_ values cited from [25].

**Table 2:** Prediction of hOCT1-3 and hMATE1/2-K DDIs for brigatinib following FDA-recommended criteria for in vivo drug–drug interaction (DDI) studies.

	Metformin	mIBG	MPP+
hOCT1			
IC_50_ (µM)	1.12 ± 0.06	17.1 ± 1.8	25.1 ± 1.4
Brigatinib unbound C_max_/IC_50_ ^1^	0.75	0.05	0.03
In vivo DDI study recommendation	Yes	No	No
hOCT2			
IC_50_ (µM)	30.2 ± 1.8	157 ± 21	>300
Brigatinib unbound C_max_/IC_50_ ^1^	0.03	0.005	<0.002
In vivo DDI study recommendation	No	No	No
hOCT3			
IC_50_ (µM)	0.85 ± 0.08	1.20 ± 0.04	2.54 ± 0.15
Brigatinib unbound C_max_/IC_50_ ^1^	0.99	0.70	0.33
In vivo DDI study recommendation	Yes	Yes	Yes
hMATE1			
IC_50_ (µM)	1.67 ± 0.15	1.93 ± 0.21	2.03 ± 0.25
Brigatinib unbound C_max_/IC_50_ ^1^	0.51	0.44	0.42
In vivo DDI study recommendation	Yes	Yes	Yes
hMATE2-K			
IC_50_ (µM)	38.3 ± 2.0	172 ± 23	>300
Brigatinib unbound C_max_/IC_50_ ^1^	0.02	0.004	<0.002
In vivo DDI study recommendation	No	No	No

^1^ Brigatinib unbound C_max_ = 0.845 µM, from the multi-discipline review of ALUNBRIG (brigatinib) by the US FDA, was used for calculations.

**Table 3:** Prediction of hOCT1-3 and hMATE1/2-K DDIs for crizotinib following FDA-recommended criteria for in vivo DDI study.

	Metformin	mIBG	MPP+
hOCT1			
IC_50_ (µM)	4.98 ± 0.20	40.3 ± 5.5 ^2^	105 ± 5
Crizotinib unbound C_max_/IC_50_ ^1^	0.02	0.002	<0.002
In vivo DDI study recommendation	No	No	No
hOCT2			
IC_50_ (µM)	6.07 ± 0.36	14.3 ± 1.7 ^2^	98.1 ± 6.4
Crizotinib unbound C_max_/IC_50_ ^1^	0.02	0.007	<0.002
In vivo DDI study recommendation	No	No	No
hOCT3			
IC_50_ (µM)	1.43 ± 0.11	2.14 ± 0.25 ^2^	6.86 ± 0.27
Crizotinib unbound C_max_/IC_50_ ^1^	0.07	0.05	0.01
In vivo DDI study recommendation	No	No	No
hMATE1			
IC_50_ (µM)	7.60 ± 0.46	6.73 ± 0.68	11.8 ± 0.7
Crizotinib unbound C_max_/IC_50_ ^1^	0.01	0.01	0.01
In vivo DDI study recommendation	No	No	No
hMATE2-K			
IC_50_ (µM)	10.1 ± 0.3	55.4 ± 3.2	125 ± 6
Crizotinib unbound C_max_/IC_50_ ^1^	0.01	0.002	<0.002
In vivo DDI study recommendation	No	No	No

^1^ Crizotinib unbound C_max_ = 0.845 µM, from the pharmacology review of XALKORI (crizotinib) by the US FDA, was used for calculations. ^2^ IC_50_ values from [25].

**Table 4:** IC_50_ values, shown as mean ± SD from three independent experiments, of brigatinib and crizotinib for hOCT2- and hMATE1-mediated [^14^C] metformin uptake after a 30-min preincubation period at 37 °C.

	IC_50_ (µM)					
	Brigatinib			Crizotinib		
	Preincubation	Coincubation	Fold Difference	Preincubation	Coincubation	Fold Difference
hOCT2	15.5 ± 0.5	36.0 ± 1.1	2.3	3.64 ± 0.29	9.30 ± 0.77	2.6
hMATE1	0.97 ± 0.09	1.41 ± 0.06	1.4	5.28 ± 0.48	7.57 ± 0.59	1.4

## Data Availability

The data generated are all included in the article.

## References

[1] Janoueix-Lerosey I., Lequin D., Brugières L., Ribeiro A., de Pontual L., Combaret V., Raynal V., Puisieux A., Schleiermacher G., Pierron G. (2008). Somatic and Germline Activating Mutations of the ALK Kinase Receptor in Neuroblastoma. Nature.

[2] Bethune G., Bethune D., Ridgway N., Xu Z. (2010). Epidermal Growth Factor Receptor (EGFR) in Lung Cancer: An Overview and Update. J. Thorac. Dis..

[3] Webb T.R., Slavish J., George R.E., Look A.T., Xue L., Jiang Q., Cui X., Rentrop W.B., Morris S.W. (2009). Anaplastic Lymphoma Kinase: Role in Cancer Pathogenesis and Small-Molecule Inhibitor Development for Therapy. Expert Rev. Anticancer Ther..

[4] Trigg R., Turner S. (2018). ALK in Neuroblastoma: Biological and Therapeutic Implications. Cancers.

[5] Chiarle R., Voena C., Ambrogio C., Piva R., Inghirami G. (2008). The Anaplastic Lymphoma Kinase in the Pathogenesis of Cancer. Nat. Rev. Cancer.

[6] Khan M., Lin J., Liao G., Tian Y., Liang Y., Li R., Liu M., Yuan Y. (2019). ALK Inhibitors in the Treatment of ALK Positive NSCLC. Front. Oncol..

[7] Chia P.L., John T., Dobrovic A., Mitchell P. (2014). Prevalence and Natural History of ALK Positive Non-Small-Cell Lung Cancer and the Clinical Impact of Targeted Therapy with ALK Inhibitors. Clin. Epidemiol..

[8] Salido M., Pijuan L., Martínez-Avilés L., Galván A.B., Cañadas I., Rovira A., Zanui M., Martínez A., Longarón R., Sole F. (2011). Increased ALK Gene Copy Number and Amplification Are Frequent in Non-Small Cell Lung Cancer. J. Thorac. Oncol..

[9] US Food and Drug Administration Crizotinib, Pharmacology Review. https://www.accessdata.fda.gov/drugsatfda_docs/nda/2011/202570Orig1s000PharmR.pdf.

[10] Krytska K., Ryles H.T., Sano R., Raman P., Infarinato N.R., Hansel T.D., Makena M.R., Song M.M., Reynolds C.P., Mossé Y.P. (2016). Crizotinib Synergizes with Chemotherapy in Preclinical Models of Neuroblastoma. Clin. Cancer Res..

[11] US National Cancer Institute Drugs Approved for Non-Small Cell Lung Cancer. https://www.cancer.gov/about-cancer/treatment/drugs/lung.

[12] Goldsmith K.C., Park J.R., Kayser K., Malvar J., Chi Y.-Y., Groshen S.G., Villablanca J.G., Krytska K., Lai L.M., Acharya P.T. (2023). Lorlatinib with or without Chemotherapy in ALK-Driven Refractory/Relapsed Neuroblastoma: Phase 1 Trial Results. Nat. Med..

[13] Giacomini K.M., Huang S.-M., Tweedie D.J., Benet L.Z., Brouwer K.L.R., Chu X., Dahlin A., Evers R., Fischer V., Hillgren K.M. (2010). Membrane Transporters in Drug Development. Nat. Rev. Drug Discov..

[14] Wagner D.J., Hu T., Wang J. (2016). Polyspecific Organic Cation Transporters and Their Impact on Drug Intracellular Levels and Pharmacodynamics. Pharmacol. Res..

[15] Li L.P., Song F.F., Weng Y.Y., Yang X., Wang K., Lei H.M., Ma J., Zhou H., Jiang H.D. (2016). Role of OCT2 and MATE1 in Renal Disposition and Toxicity of Nitidine Chloride. Br. J. Pharmacol..

[16] Sata R., Ohtani H., Tsujimoto M., Murakami H., Koyabu N., Nakamura T., Uchiumi T., Kuwano M., Nagata H., Tsukimori K. (2005). Functional Analysis of Organic Cation Transporter 3 Expressed in Human Placenta. J. Pharmacol. Exp. Ther..

[17] Lee N., Duan H., Hebert M.F., Liang C.J., Rice K.M., Wang J. (2014). Taste of a Pill. J. Biol. Chem..

[18] Koepsell H., Lips K., Volk C. (2007). Polyspecific Organic Cation Transporters: Structure, Function, Physiological Roles, and Biopharmaceutical Implications. Pharm. Res..

[19] Kekuda R., Prasad P.D., Wu X., Wang H., Fei Y.-J., Leibach F.H., Ganapathy V. (1998). Cloning and Functional Characterization of a Potential-Sensitive, Polyspecific Organic Cation Transporter (OCT3) Most Abundantly Expressed in Placenta. J. Biol. Chem..

[20] Duan H., Wang J. (2010). Selective Transport of Monoamine Neurotransmitters by Human Plasma Membrane Monoamine Transporter and Organic Cation Transporter 3. J. Pharmacol. Exp. Ther..

[21] Lee N., Hebert M.F., Wagner D.J., Easterling T.R., Liang C.J., Rice K., Wang J. (2018). Organic Cation Transporter 3 Facilitates Fetal Exposure to Metformin during Pregnancy. Mol. Pharmacol..

[22] Zhang S., Lovejoy K.S., Shima J.E., Lagpacan L.L., Shu Y., Lapuk A., Chen Y., Komori T., Gray J.W., Chen X. (2006). Organic Cation Transporters Are Determinants of Oxaliplatin Cytotoxicity. Cancer Res..

[23] Huang K.M., Zavorka Thomas M., Magdy T., Eisenmann E.D., Uddin M.E., DiGiacomo D.F., Pan A., Keiser M., Otter M., Xia S.H. (2021). Targeting OCT3 Attenuates Doxorubicin-Induced Cardiac Injury. Proc. Natl. Acad. Sci. USA.

[24] Hucke A., Ciarimboli G. (2016). The Role of Transporters in the Toxicity of Chemotherapeutic Drugs: Focus on Transporters for Organic Cations. J. Clin. Pharmacol..

[25] López Quiñones A.J., Wagner D.J., Wang J. (2020). Characterization of *Meta* -Iodobenzylguanidine (MIBG) Transport by Polyspecific Organic Cation Transporters: Implication for MIBG Therapy. Mol. Pharmacol..

[26] Masereeuw R., Russel F.G.M. (2001). Mechanisms and Clinical Implications of Renal Drug Excretion. Drug Metab. Rev..

[27] Morrissey K.M., Stocker S.L., Wittwer M.B., Xu L., Giacomini K.M. (2013). Renal Transporters in Drug Development. Annu. Rev. Pharmacol. Toxicol..

[28] Koepsell H. (2015). Role of Organic Cation Transporters in Drug–Drug Interaction. Expert Opin. Drug Metab. Toxicol..

[29] US Food and Drug Adminstration In Vitro Drug Interaction Studies—Cytochrome P450 Enzyme- and Transporter-Mediated Drug Interactions Guidance for Industry. https://www.fda.gov/regulatory-information/search-fda-guidance-documents/in-vitro-drug-interaction-studies-cytochrome-p450-enzyme-and-transporter-mediated-drug-interactions.

[30] Koepsell H. (2019). Multiple Binding Sites in Organic Cation Transporters Require Sophisticated Procedures to Identify Interactions of Novel Drugs. Biol. Chem..

[31] Yin J., Duan H., Wang J. (2016). Impact of Substrate-Dependent Inhibition on Renal Organic Cation Transporters HOCT2 and HMATE1/2-K-Mediated Drug Transport and Intracellular Accumulation. J. Pharmacol. Exp. Ther..

[32] Martínez-Guerrero L.J., Wright S.H. (2013). Substrate-Dependent Inhibition of Human MATE1 by Cationic Ionic Liquids. J. Pharmacol. Exp. Ther..

[33] Martínez-Guerrero L.J., Morales M., Ekins S., Wright S.H. (2016). Lack of Influence of Substrate on Ligand Interaction with the Human Multidrug and Toxin Extruder, MATE1. Mol. Pharmacol..

[34] Uddin M.E., Garrison D.A., Kim K., Jin Y., Eisenmann E.D., Huang K.M., Gibson A.A., Hu Z., Sparreboom A., Hu S. (2021). Influence of YES1 Kinase and Tyrosine Phosphorylation on the Activity of OCT1. Front. Pharmacol..

[35] Alim K., Moreau A., Bruyère A., Jouan E., Denizot C., Nies A.T., Parmentier Y., Fardel O. (2021). Inhibition of Organic Cation Transporter 3 Activity by Tyrosine Kinase Inhibitors. Fundam. Clin. Pharmacol..

[36] Minematsu T., Giacomini K.M. (2011). Interactions of Tyrosine Kinase Inhibitors with Organic Cation Transporters and Multidrug and Toxic Compound Extrusion Proteins. Mol. Cancer Ther..

[37] Infarinato N.R., Park J.H., Krytska K., Ryles H.T., Sano R., Szigety K.M., Li Y., Zou H.Y., Lee N.V., Smeal T. (2016). The ALK/ROS1 Inhibitor PF-06463922 Overcomes Primary Resistance to Crizotinib in ALK-Driven Neuroblastoma. Cancer Discov..

[38] Siaw J.T., Wan H., Pfeifer K., Rivera V.M., Guan J., Palmer R.H., Hallberg B. (2016). Brigatinib, an Anaplastic Lymphoma Kinase Inhibitor, Abrogates Activity and Growth in ALK-Positive Neuroblastoma Cells, *Drosophila* and Mice. Oncotarget.

[39] Nalluri S., Peirce S.K., Tanos R., Abdella H.A., Karmali D., Hogarty M.D., Goldsmith K.C. (2015). EGFR Signaling Defines Mcl^−1^ Survival Dependency in Neuroblastoma. Cancer Biol. Ther..

[40] Amundsen R., Christensen H., Zabihyan B., Åsberg A. (2010). Cyclosporine A, but Not Tacrolimus, Shows Relevant Inhibition of Organic Anion-Transporting Protein 1B1-Mediated Transport of Atorvastatin. Drug Metab. Dispos..

[41] Gertz M., Cartwright C.M., Hobbs M.J., Kenworthy K.E., Rowland M., Houston J.B., Galetin A. (2013). Cyclosporine Inhibition of Hepatic and Intestinal CYP3A4, Uptake and Efflux Transporters: Application of PBPK Modeling in the Assessment of Drug-Drug Interaction Potential. Pharm. Res..

[42] Coleman R.E., Stubbs J.B., Barrett J.A., de la Guardia M., LaFrance N., Babich J.W. (2009). Radiation Dosimetry, Pharmacokinetics, and Safety of Ultratrace^TM^ Iobenguane I-131 in Patients with Malignant Pheochromocytoma/Paraganglioma or Metastatic Carcinoid. Cancer Biother. Radiopharm..

[43] Chin B.B., Kronauge J.F., Femia F.J., Chen J., Maresca K.P., Hillier S., Petry N.A., James O.G., Oldan J.D., Armor T. (2014). Phase-1 Clinical Trial Results of High-Specific-Activity Carrier-Free^123^ I-Iobenguane. J. Nucl. Med..

[44] Lopez Quinones A., Salvador-Vieira L., Wang J. (2021). Organic Cation Transporter 3 Mediates Tissue Uptake and Accumulation of Meta-Iodobenzylguanidine (MIBG). FASEB J..

[45] Bayer M., Schmitt J., Dittmann H., Handgretinger R., Bruchelt G., Sauter A.W. (2016). Improved Selectivity of MIBG Uptake into Neuroblastoma Cells in Vitro and in Vivo by Inhibition of Organic Cation Transporter 3 Uptake Using Clinically Approved Corticosteroids. Nucl. Med. Biol..

[46] López Quiñones A.J., Vieira L.S., Wang J. (2022). Clinical Applications and the Roles of Transporters in Disposition, Tumor Targeting, and Tissue Toxicity of *meta*-Iodobenzylguanidine. Drug Metab. Dispos..

[47] Streby K.A., Shah N., Ranalli M.A., Kunkler A., Cripe T.P. (2015). Nothing but NET: A Review of Norepinephrine Transporter Expression and Efficacy of ^131^ I-mIBG Therapy. Pediatr. Blood Cancer.

[48] Hemminki K., Li X., Sundquist J., Sundquist K. (2010). Risk of Cancer Following Hospitalization for Type 2 Diabetes. Oncologist.

[49] Chen H., Yao W., Chu Q., Han R., Wang Y., Sun J., Wang D., Wang Y., Cao M., He Y. (2015). Synergistic Effects of Metformin in Combination with EGFR-TKI in the Treatment of Patients with Advanced Non-Small Cell Lung Cancer and Type 2 Diabetes. Cancer Lett..

[50] Gong L., Goswami S., Giacomini K.M., Altman R.B., Klein T.E. (2012). Metformin Pathways. Pharmacogenet. Genom..

[51] Hundal R.S., Krssak M., Dufour S., Laurent D., Lebon V., Chandramouli V., Inzucchi S.E., Schumann W.C., Petersen K.F., Landau B.R. (2000). Mechanism by Which Metformin Reduces Glucose Production in Type 2 Diabetes. Diabetes.

[52] Gorbunov D., Gorboulev V., Shatskaya N., Mueller T., Bamberg E., Friedrich T., Koepsell H. (2008). High-Affinity Cation Binding to Organic Cation Transporter 1 Induces Movement of Helix 11 and Blocks Transport after Mutations in a Modeled Interaction Domain between Two Helices. Mol. Pharmacol..

[53] Minuesa G., Volk C., Molina-Arcas M., Gorboulev V., Erkizia I., Arndt P., Clotet B., Pastor-Anglada M., Koepsell H., Martinez-Picado J. (2009). Transport of Lamivudine [(-)-β-l-2′,3′-Dideoxy-3′-Thiacytidine] and High-Affinity Interaction of Nucleoside Reverse Transcriptase Inhibitors with Human Organic Cation Transporters 1, 2, and 3. J. Pharmacol. Exp. Ther..

[54] Tátrai P., Schweigler P., Poller B., Domange N., de Wilde R., Hanna I., Gáborik Z., Huth F. (2019). A Systematic In Vitro Investigation of the Inhibitor Preincubation Effect on Multiple Classes of Clinically Relevant Transporters. Drug Metab. Dispos..

